# Protective effect of *Asarum* extract in rats with adjuvant arthritis

**DOI:** 10.3892/etm.2014.1941

**Published:** 2014-08-29

**Authors:** WENQIANG ZHANG, JUAN ZHANG, MING ZHANG, LIN NIE

**Affiliations:** 1 Department of Orthopedics, Shandong Qianfoshan Hospital Affiliated to Shandong University, Jinan, Shandong 250014, P.R. China; 2 Department of Cardiology, No. 2 Hospital Affiliated to Shandong University, Jinan, Shandong 250033, P.R. China; 3 Department of Orthopedics, Qilu Hospital Affiliated to Shandong University, Jinan, Shandong 250012, P.R. China

**Keywords:** adjuvant arthritis, rheumatoid arthritis

## Abstract

The aim of the present study was to investigate the protective effect of *Asarum* extract on rats with adjuvant arthritis (AA) and to determine the underlying mechanism. An AA model was established by injecting Freund’s complete adjuvant into the rats. The degree of toe swelling, arthritis index, spleen index, and the expression levels of tumor necrosis factor (TNF)-α, interleukin (IL)-1β and IL-6 were measured. In addition, the underlying molecular mechanism was investigated using murine macrophage-derived RAW 264.7 cells. *Asarum* extract was found to significantly reduce the severity of arthritis by decreasing hind paw swelling, the arthritis index, the spleen index, and TNF-α, IL-1β and IL-6 expression levels in plasma. *In vitro*, *Asarum* extract inhibited the nuclear factor (NF)-κB and mitogen-activated protein kinase (MAPK) signaling pathways. These results indicate that *Asarum* extract may be a therapeutic agent for AA and may exert an anti-inflammatory effect by mediating the NF-κB and MAPK signaling pathways.

## Introduction

Rheumatoid arthritis (RA) is a chronic, disabling and systemic autoimmune disease that leads to joint inflammation, as well as progressive cartilage and bone erosion (1–3). RA also causes tissue inflammation around the joints and other organs of the body. The disease affects up to 1% of the adult population worldwide (4).

Freund’s complete adjuvant (FCA)-induced arthritis shares a number of characteristics with RA (5). FCA mirrors the pathology of RA, by causing hyperplasia of the synovial tissues, inflammatory infiltration of the joints, and the destruction of bone and cartilage.

*Asarum*, a traditional Chinese medicine known as ‘xixin’, is widely distributed in the north-east of China (6). The herb has been used for the treatment of colds, and as an analgesic, antitussive or anti-allergic remedy. Modern pharmacological studies have shown that *Asarum* species exhibit anti-inflammatory, antitussive, anti-allergic, anti-hyperlipidemic and anti-myocardiac ischemia properties by enhancing myocardial contractility, antiarrhythmic activities and other mechanisms (7,8).

In the present study, the anti-arthritic activity of *Asarum* extracts in rats with FCA-induced adjuvant arthritis was evaluated and the anti-inflammatory mechanisms in lipopolysaccharide (LPS)-treated RAW 264.7 macrophages were explored. The underlying mechanism was also investigated.

## Materials and methods

### Materials

Unless otherwise specified, all chemicals were purchased from Sigma-Aldrich (St. Louis, MO, USA). Dulbecco’s modified Eagle’s medium (DMEM), fetal bovine serum and the antibiotic-antimycotic solution were purchased from Gibco (Auckland, New Zealand). The ELISA kits for TNF-α, IL-1β and IL-6 were obtained from Neobioscience (Beijing, China). Primary antibodies against extracellular signal-regulated kinase (ERK), phospho-ERK, p38, phospho-p38, c-Jun N-terminal kinase (JNK), phospho-JNK, IκB-α, phospho-IκB-α, p65, phospho-p65, IKKβ and phospho-IKKβ were purchased from Cell Signaling Technology (Beverly, MA, USA). Horseradish peroxidase-conjugated secondary antibodies were also obtained from Cell Signaling Technology.

### Induction of adjuvant arthritis (AA)

Sprague-Dawley rats (250–300 g; obtained from Vital River Laboratories, Beijing, China) were maintained under conditions of standard lighting (an alternating 12 h light/dark cycle), temperature (23–25°C) and humidity (40–70%). The rats were immunized (day 0) with a single intradermal injection of 0.1 ml FCA into the right hind paw. FCA was prepared by mixing 10 mg heat-inactivated (58°C, 1 h) Bacillus Calmette-Guérin (BCG) with 1 ml sterile paraffin oil. Control animals received 0.1 ml saline (0.9% NaCl solution). This study was performed in accordance with the recommendations from the Guide for the Care and Use of Laboratory Animals by the National Institutes of Health [Eighth Edition (2011); Bethesda, MD, USA]. The animal use protocol was reviewed and approved by the Institutional Animal Care and Use Committee (IACUC) of Shandong University (Jinan, China).

### Grouping

Treatment with the test agent began on day 7. All treatments were orally administered to the rats. Rats were divided into six groups: Group 1, normal control; Group 2, arthritis control; Group 3, glycoside of *Tripterygium wilfordii* (GTW) 40 mg/kg/day; Group 4, *Asarum* extract-low dose (L) 20 mg/kg/day; Group 5, *Asarum* extract-medium dose (M) 40 mg/kg/day; and Group 6, *Asarum* extract-high dose (H) 80 mg/kg/day. GTW was purchased from Shanghai Fudan Forward Pharmaceutical Co., Ltd. (Shanghai, China). *Ansarum* extract was extracted and prepared in the Medical Experimental Center of Shandong Qianfoshan Hospital (Jinan, Shandong).

### Evaluation of AA

Paw volumes were recorded on days 14, 18, 22, 26 and 30. The arthritis index (AI) was classified using a five-value scale: 0, no swelling; 1 point, swelling on the joint of the little toe; 2 points: swelling on the metatarsal phalange joint and foot; 3 points, swelling on the hind paw excluding the ankle; and 4 points, swelling on the hind paw and ankle. The sum of points for each rat was then calculated.

The hind paw volume (ml) of all animal groups was measured by a plethysmometer on days 14, 18, 22, 26 and 30 after the injection of FCA emulsion. The paw swelling rate (%) was expressed as increased multiples of right hind paw volume by subtraction of the basic paw volume, as a proportion of the basic paw volume.

The ratio of spleen weight to rat body weight represented the spleen index. Blood was collected from the retro-orbital plexus for measurement of biochemical and hematological parameters, namely tumor necrosis factor (TNF)-α, interleukin (IL)-1β and IL-6.

### Cell cultures

The RAW 264.7 murine macrophage/monocyte cell line was maintained at 37°C and at 5% CO_2_ in Dulbecco’s modified Eagle’s medium (Gibco-BRL, Gaithersburg, MD, USA) with 10% fetal bovine serum (Gibco-BRL). The RAW 264.7 cells were plated at a density of 2.0×10^5^ cells/well and incubated overnight. *Asarum* extracts (2, 10 and 50 μM) or aspirin (50 μM) were then added. Following 2 h of exposure, lipopolysaccharide (LPS) was added to the treated cells at a final concentration of 0.5 μg/ml. The resultant cell lysates were immunoblotted using affinity-purified antibodies against IKKβ, phospho-IKKβ, IκB, phospho-IκB, P65, P65, phospho-P65, phospho-ERK and phospho-JNK (9,10).

### Western blot analysis

Following treatment, the cells were washed three times with phosphate-buffered saline (PBS), transferred into a 100 μl loading buffer [10 mm Tris-HCl, pH 6.8, glycerol 2%, bromophenol blue 2%, sodium dodecyl sulfate (SDS) 0.4% and mercaptoethanol 0.14%] and incubated on ice for 30 min. For western blotting, the protein of the cell lysate was separated in 10% SDS-PAGE gel and transferred onto nitrocellulose membranes. After blocking with 5% bovine serum albumin (BSA) for 2 h at room temperature, the membranes were washed three times with Tris-HCl buffer solution containing Tween-20 (TBST). The membranes were then incubated with the primary antibodies overnight at 4°C. The membranes were washed and then incubated with horseradish peroxidase-conjugated secondary mouse or rabbit antibodies. The BSA and antibodies were suspended in TBST.

### Statistical analysis

Values are presented as the mean ± standard deviation. Statistical analysis was performed using a two-tailed Student’s t-test, and P<0.05 was considered to indicate a statistically significant difference. Calculations were performed by using SPSS software, version 13.0 (SPSS, Inc., Chicago, IL, USA).

## Results

### AI

From day 14, a statistically significant (P<0.05) increase in AI was observed in the FCA-induced arthritic animals in the disease control group compared the normal control group, as in previous study (11). Treatment with *Asarum* extract at 40 and 80 mg/kg yielded significant (P<0.05) reductions in the swelling pain scores compared with those in the disease control group after 30 days, as shown in Table I.

### Rate of swelling

Table II shows the rate of swelling. Right hind paw swelling was found to be significantly increased in AA rats compared with normal animals. *Asarum* extract at 40 and 80 mg/kg diminished the rate of swelling.

### Spleen index

**Figure 1:**
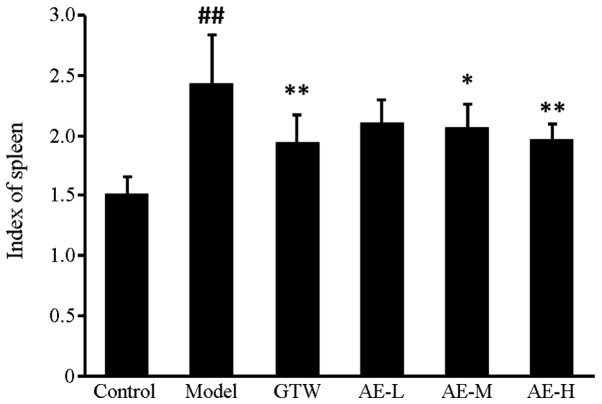
Effects of *Asarum* extracts (AE) on the spleen index of rats with adjuvant-induced arthritis. Values are expressed as the mean ± standard deviation ^##^P<0.01. versus control; ^*^P<0.05, ^**^P<0.01, versus model. GTW, glycoside of *Tripterygium wilfordii*; L, low dose (20 mg/kg); M, medium dose (40 mg/kg); H, high dose (80 mg/kg).

A significant (P<0.01) increase in the spleen index of the FCA-induced arthritic animals was observed in the disease control group compared with the normal control group. Administration of *Asarum* extract caused a reduction in the spleen index of FCA-induced arthritic rats. Specifically, *Asarum* extract at 40 and 80 mg/kg significantly decreased the spleen index compared with that in the disease control group (P<0.05, P<0.01; Fig. 1).

### IL-1β, IL-6 and TNF-α expression levels

IL-1β, IL-6 and TNF-α expression levels were detected using a standard ELISA (12–14), and are presented in Table III. Rats with FCA-induced arthritis had significantly (P<0.01) increased IL-1β, IL-6 and TNF-α expression levels compared with those in the normal control group. However, administration of *Asarum* extract or GTW caused significant reductions in the IL-1β, IL-6 and TNF-α expression levels.

### Mitogen-activated protein kinase (MAPK) signaling pathway

**Figure 2:**
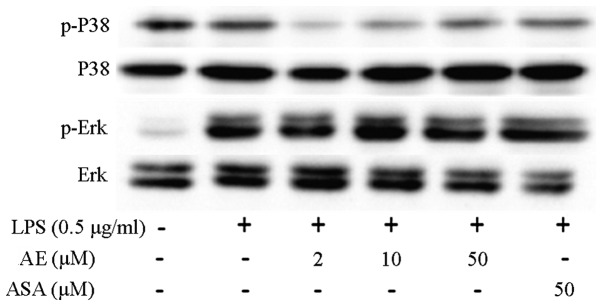
Inhibitory effect of *Asarum* extracts on LPS-stimulated phosphorylation of MAPKs in RAW 264.7 cells. The cells were pretreated with *Asarum* extracts followed by incubation with LPS (0.5 μg/ml) for an additional 15 min. Total cell lysates were then prepared for detection of P-p38/ERK MAPKs by Western blot analysis using specific antibodies. AE, *Ansarum* extracts; LPS, lipopolysaccharide; ASA, aspirin; MAPK, mitogen-activated protein kinase; ERK, extracellular signal-regulated kinase; P, phosphorylated.

Western blot analysis was performed to evaluate the phosphorylation of MAPK (15,16). Fig. 2 demonstrates that the expression of phosphorylated ERK and p38-MAPK increased in the LPS-induced RAW 264.7 cells. *Asarum* extract (10 and 50 μM) significantly decreased the phosphorylation of ERK and p38, but not JNK (data not shown). Aspirin administration did not cause significant changes in the phosphorylation levels of ERK and p38.

### Nuclear factor (NF)-κB signaling pathway

**Figure 3:**
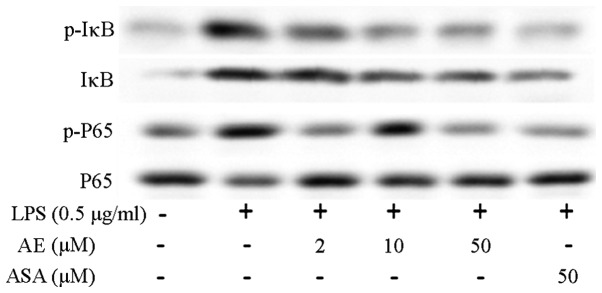
Inhibitory effects of *Asarum* extracts on LPS-stimulated phosphorylation of nuclear factor-κB in RAW 264.7 cells. The cells were pretreated with *Asarum* extracts followed by incubation with LPS (0.5 μg/ml) for an additional 15 min. Total cell lysates were then prepared in order to determine p-IκB/P65 expression levels using western blot analysis with specific antibodies. AE, *Ansarum* extracts; LPS, lipopolysaccharide; ASA, aspirin; P, phosphorylated.

Phosphorylated IκB and P65 expression levels increased in LPS-induced RAW264.7 cells (16–18). *Asarum* extract at 2, 10 and 50 μM significantly decreased the phosphorylation of IκB and P65. Aspirin had no significant effect on the phosphorylation of IκB and P65 (Fig. 3).

## Discussion

RA is an autoimmune disease characterized by multi-joint arthritis and joint erosion, which involves inflammation and immunity, and may be inherited. However, the mechanisms of RA have not been clearly elucidated. AA is an experimental model induced by BCG that is recognized as 65 kDa anti-heat shock protein (HSP65). HSP65 is similar to the conservative sequences in AA rat auto-antigens as HSP65 introduces an autoimmune response by activating T-cell clones. Therefore, AA model rats, which have characteristics similar to those of RA, are the ideal animal model for RA (19).

Joint swelling and pain are the initial manifestations of RA. The podarthrum of the arthritis group animals revealed swelling following injection, and symptoms developed with time. Disease severity was evaluated objectively by measuring the toe volume and by grading the swelling score. Fourteen days following model establishment, the animals in the GTW and all the *Asarum* extract groups demonstrated an alleviation of secondary symptoms to varying degrees. Toe volume and swelling score decreased, indicating an improvement in AA rats.

The AA model is an immune hyperfunctional model, and spleen hyperplasia has been previously reported in AA model rats (20). In the present study, 40 mg/kg/day GTW and 40 or 80 mg/kg/day *Asarum* extract were found to significantly reduce spleen hyperplasia (P<0.05, versus model group); however, spleen hyperplasia remained higher compared with that in normal animals. This finding suggests that *Asarum* extract may help in the recovery of the hyperfunctioning of immune organs without causing damage.

Numerous cytokines, including TNF-α, IL-6 and IL-1β, are released in RA and have an important role in the inflammation and activation of synoviocytes, which induce joint injury (21). IL-1β is an important pro-inflammatory cytokine that regulates immunity and inflammation. IL-1β increases in acute inflammation and induces damage to cells and tissues. Another pro-inflammatory cytokine, TNF-α, is produced by monocytes and macrophages, as well as by T lymphocytes and neutrophils. Release of TNF-α not only causes an inflammatory response, but also induces the production of IL-1β and IL-6 by macrophages, thereby aggravating local inflammatory responses. IL-6 transforms B lymphocyte precursors into antibodies, thus producing mature B lymphocytes, promoting growth and differentiation of bone marrow cells in cooperation with colony stimulating factors and improving the degradation function of natural killer cells (22). In the present study, the expression levels of TNF-α, IL-6 and IL-1β in the plasma of the model animals were higher compared with those in normal animals, whilst *Asarum* extract decreased serum inflammatory cytokines to varying degrees. These results indicate that *Asarum* extract reduces the inflammatory response of AA model rats by inhibiting pro-inflammatory factors, which in turn relieve joint damage.

Numerous cytokines and inflammatory factors are involved in the inflammatory response and have various roles through different pathways, including the MAPK and NF-κB pathways (23–25). The nuclear transcription factor NF-κB is involved in inflammation. During the resting condition, NF-κB is inhibited by the binding of IκB and remains in the cytoplasm in default mode. IκB is a p65/p50 inhibitor, and stimulation through LPS induces phosphorylation of IκB kinase, leading to polyubiquitination and degradation of IκB, as well as the release of p65/60 protein. P65/60 protein moves into the cell nucleus and binds to DNA, activating gene transcription and producing multiple inflammatory factors that are involved in RA. The MAPK signaling pathway regulates the gene expression of a number of cytokines, chemotactic factors, growth factors, adherence factors and other enzymes. This pathway also participates in immune and inflammation reactions and has a vital role in cell proliferation, differentiation and apoptosis (26). The results obtained in the present study demonstrated that *Asarum* extract significantly increases the phosphorylation of IKKβ, IκB and p65, which results in the activation of the NF-κB signaling pathway. *Asarum* extract was also found to significantly inhibit the phosphorylation of P38 and ERK, thereby blocking the activation of the MAPK signaling pathway. The combined inhibition of the two pathways may prevent the inflammatory response, which may be the mechanism by which *Asarum* extract inhibits AA progression.

These findings indicate that *Asarum* extract is a potential therapeutic agent for AA as it exerts an anti-inflammatory effect by mediating the NF-κB and MAPK signaling pathways.

## Figures and Tables

**Table I:** Effect of *Asarum* extracts on swelling scores in rats with adjuvant-induced arthritis (mean ± standard deviation).

		Scores				
		
Groups	n	Day 14	Day 18	Day 22	Day 26	Day 30
Control	12	0	0	0	0	0
Model	12	0.67±0.65	1.25±0.75	2.5±1.51	2.42±1.51	2.25±1.22
GTW, 40 mg/kg	12	0.58±0.67	1±0.85	1.42±1.31	1.25±1.22^b^	1.17±0.94^b^
*Asarum* extract						
20 mg/kg	12	0.67±0.65	1.17±0.58	1.67±0.89	1.67±1.15	1.42±0.99
40 mg/kg	12	0.58±0.67	1.08±0.79	1.58±1.08	1.5±1.17	1.33±0.89^b^
80 mg/kg	12	0.58±0.67	1±0.60	1.5±1	1.33±0.98^b^	1.25±0.87^b^

a P<0.01 versus control group;

b P<0.05 and

c P<0.01, versus model group.

GTW, glycoside of *Tripterygium wilfordii*.

**Table II:** Effect of *Asarum* extract on the rate of swelling in rats with adjuvant-induced arthritis (mean ± standard deviation).

		Rate of swelling (%)				
		
Groups	n	Day 14	Day 18	Day 22	Day 26	Day 30
Control	12	1.10±2.41	2.21±1.75	1.38±3.13	2.98±2.39	0.14±1.44
Model	12	70.73±12.08^a^	79.28±15.17^a^	72.25±15.37^a^	70.08±13.38^a^	66.72±11.90^a^
GTW, 40 mg/kg	12	66.26±13.02	70.40±12.93	62.33±9.76	57.09±11.36^b^	53.26±11.42^c^
*Asarum* extract						
20 mg/kg	12	66.05±11.68	73.19±13.84	66.96±14.38	60.59±12.50	58.27±8.05
40 mg/kg	12	65.92±13.15	72.82±9.90	66.78±11.12	61.79±11.92	56.74±8.19^b^
80 mg/kg	12	67.98±11.46	71.87±12.79	62.61±12.11	56.70±11.54^b^	54.36±10.71^b^

a P<0.01, versus control group;

b P<0.05 and

c P<0.01, versus model group.

GTW, glycoside of *Tripterygium wilfordii*.

**Table III:** Effects of *Asarum* extracts on IL-1β, IL-6 and TNF-α in rats with adjuvant-induced arthritis (mean ± standard deviation).

Groups	n	TNF-α (pg/ml)	IL-6 (pg/ml)	IL-1β (pg/ml)
Control	12	69.5±8.86	73.4±10.32	14.225±3.98
Model	12	275.54±45.07^a^	139.56±26.29^a^	89.01±10.73^a^
GTW, 40 mg/kg	12	186.01±34.03^c^	86.58±10.13^c^	60.43±14.44^c^
*Asarum* extract				
20 mg/kg	12	259.08±32.10	119.68±25.67	81.4±10.15
40 mg/kg	12	224.21±52.02^c^	107.73±31.03^c^	75.23±19.99^c^
80 mg/kg	12	192.49±26.16^c^	95.8±23.74^c^	64.58±13.00^c^

a P<0.01 versus control;

b P<0.05 and

c P<0.01 versus model.

TNF-α, tumor necrosis factor; IL, interleukin; GTW, glycoside of *Tripterygium wilfordii*.
